# Quantitative Morphometry and Machine Learning Model to Explore Duodenal and Rectal Mucosal Tissue of Children with Environmental Enteric Dysfunction

**DOI:** 10.4269/ajtmh.22-0063

**Published:** 2023-03-13

**Authors:** Marium Khan, Zehra Jamil, Lubaina Ehsan, Fatima Zulqarnain, Sanjana Srivastava, Saman Siddiqui, Philip Fernandes, Muhammad Raghib, Saurav Sengupta, Zia Mujahid, Zubair Ahmed, Romana Idrees, Sheraz Ahmed, Fayaz Umrani, Najeeha Iqbal, Christopher Moskaluk, Shyam Raghavan, Lin Cheng, Sean Moore, Syed Asad Ali, Junaid Iqbal, Sana Syed

**Affiliations:** ^1^ Department of Pediatrics, School of Medicine, University of Virginia, Charlottesville, Virginia;; ^2^ Department of Biological and Biomedical Sciences, Aga Khan University, Karachi, Pakistan;; ^3^ Department of Pediatrics and Child Health, Aga Khan University, Karachi, Pakistan;; ^4^ School of Data Science, University of Virginia, Charlottesville, Virginia;; ^5^ Department of Pathology and Laboratory Medicine, Aga Khan University, Karachi, Pakistan;; ^6^ Department of Pathology, University of Virginia, Charlottesville, Virginia;; ^7^ Department of Pathology, Rush University, Chicago, Illinois;; ^8^ Department of Public Health Sciences, School of Medicine, University of Virginia, Charlottesville, Virginia

## Abstract

Environmental enteric dysfunction (EED) is a subclinical enteropathy prevalent in resource-limited settings, hypothesized to be a consequence of chronic exposure to environmental enteropathogens, resulting in malnutrition, growth failure, neurocognitive delays, and oral vaccine failure. This study explored the duodenal and colonic tissues of children with EED, celiac disease, and other enteropathies using quantitative mucosal morphometry, histopathologic scoring indices, and machine learning–based image analysis from archival and prospective cohorts of children from Pakistan and the United States. We observed villus blunting as being more prominent in celiac disease than in EED, as shorter lengths of villi were observed in patients with celiac disease from Pakistan than in those from the United States, with median (interquartile range) lengths of 81 (73, 127) µm and 209 (188, 266) µm, respectively. Additionally, per the Marsh scoring method, celiac disease histologic severity was increased in the cohorts from Pakistan. Goblet cell depletion and increased intraepithelial lymphocytes were features of EED and celiac disease. Interestingly, the rectal tissue from cases with EED showed increased mononuclear inflammatory cells and intraepithelial lymphocytes in the crypts compared with controls. Increased neutrophils in the rectal crypt epithelium were also significantly associated with increased EED histologic severity scores in duodenal tissue. We observed an overlap between diseased and healthy duodenal tissue upon leveraging machine learning image analysis. We conclude that EED comprises a spectrum of inflammation in the duodenum, as previously described, and the rectal mucosa, warranting the examination of both anatomic regions in our efforts to understand and manage EED.

## INTRODUCTION

Environmental enteric dysfunction (EED) is a chronic, inflammatory, subclinical condition prevalent in low- and middle-income countries (LMICs)^1^ that is hypothesized to be a consequence of persistent environmental exposure to enteropathogens.^2^ Environmental enteric dysfunction is characterized by morphologic changes including intestinal mucosal inflammation and villus blunting, as well as functional damage, including altered gut permeability, and reduced intestinal absorption.^3^ Diminished intestinal absorption leads to growth faltering, neurodevelopmental disability, and poor response to oral vaccines and nutritional therapy.^4^^,^^5^ The persistence of stark nutritional disparities in LMIC settings versus their high-income counterparts directly impacts childhood growth rates, which may be secondary to defects in intestinal absorptive capacity due to the abovementioned environmental factors.^6^ Although health interventions are being deployed worldwide to tackle malnutrition, there is great disparity between the responses to these interventions in high-income and LMIC settings because of the prevalence of EED in resource-poor areas.^2^^,^^6^ Moreover, current efforts to establish diagnostic biomarkers for EED, as well as effective interventions to treat or prevent this enteropathy, are in their infancy.^7^^,^^8^

As with other enteropathies, the current gold standard for the diagnosis of EED, as well as the evaluation of EED pathophysiology, is the histopathologic examination of duodenal biopsies. Upon evaluation of children with EED across multiple geographic sites, the EED histologic scoring index was developed to quantify the extent of morphologic changes associated with EED and is a step toward a better characterization of EED as an inflammatory enteropathy on a spectrum of severity.^9^ Environmental enteric dysfunction shares histopathologic features with celiac disease, such as altered villus-to-crypt ratios, increased intraepithelial T cells, lamina propria T-cell infiltrates, and B-cell aggregates.^6^ Kelly et al.^10^ reported a correlation between villus height and markers of intestinal permeability among an adult population in Zambia. Additionally, our group previously reported the presence of goblet cells and intraepithelial lymphocytes (IELs) as being key distinguishing features of EED.^11^^,^^12^ Additionally, studies show that the EED transcriptome exhibits the suppression of antioxidant and detoxification genes and the induction of antimicrobial response genes similar to celiac disease.^13^ In addition to duodenal inflammation, previous studies have implied that EED may also be associated with colonic mucosal and histomorphologic alterations.^14^^,^^15^ However, there is a dearth of data available to understand whether there is an environmental colonopathy partnering with EED.

In this study, we present the evaluation of small bowel enteropathy in children with EED using histomorphometry and machine learning–based biopsy image analysis methods and compare our results with those of patients with celiac disease and controls. We also characterize differences in celiac disease across geography. Furthermore, we had the unique opportunity to explore the prevalence of colonopathy in this population by evaluating alterations in the colonic mucosa in children with EED.

## MATERIALS AND METHODS

### Study design and selection of cohorts.

This study used retrospective, archival samples from two different sites (Pakistan and the United States) and the prospective collection of samples from Pakistani children with suspected EED (Supplemental Figure 1). From the Pakistani site, clinical metadata and archival duodenal histopathology slides (Department of Pathology, Aga Khan University [AKU]) were collected from children under the age of 5 years who presented with abdominal pain, poor growth, diarrhea, and/or blood in stools and subsequently underwent esophagogastroduodenoscopy or colonoscopy with biopsy between 2007 and 2017. These slides were classified using prior clinical histopathologic evaluation into three categories: 1) celiac disease, 2) chronic duodenitis, and 3) histopathologically undiagnosed (based on the absence of any gastrointestinal diseases on tissue biopsy) after assessment by two institutional clinical pathologists.

Slides from the U.S. cohort were obtained from the clinical archives of the University of Virginia (UVA), in Charlottesville, VA, between 1992 and 2017 from children under 18 years of age who had duodenal and rectal biopsies available. Clinical diagnosis of celiac disease was considered a disease comparison group, whereas those with no histopathologic abnormalities on biopsy were selected as controls. Similarly, for rectal biopsy, clinical confirmation of ulcerative proctitis/colitis or cryptitis was chosen as the disease comparison group, and patients with no histopathologic abnormalities were selected as controls. Archival slides from patients with other gastrointestinal diseases (eosinophilic esophagitis, gastritis, inflammatory bowel disease) or slides with artifacts obscuring duodenal tissue were excluded from this analysis.

Informed consent was obtained from the parents of the children for whom archival tissue blocks were used for analysis. This study was approved by the AKU Ethical Review Committee (ERC#3836-Ped-ERC-15) and the UVA Institutional Review Board (HSR IRB# 20107). Data used for the pretrained image analysis platform have been described elsewhere.^12^^,^^16^

To explore causes of malnutrition, the samples from patients with EED in Pakistan included prospectively obtained duodenal biopsies from children refractory to nutritional intervention who were enrolled in the Study of Environmental Enteropathy and Malnutrition.^17^ The enrollment procedures and patient selection for endoscopic evaluation of EED have been published elsewhere.^18^ A subset of patients diagnosed with EED also underwent flexible sigmoidoscopy with rectal biopsy if they had persistent diarrhea and/or blood in the stool.

Hematoxylin-eosin (H&E)–stained biopsy glass slides were digitized at ×40 magnification using the Olympus VS120 scanner (Olympus Corporation Inc., Center Valley, PA) at AKU and the Leica SCN400 brightfield scanner (Leica Microsystems CMS GmbH, Mannheim, Germany) at UVA.

### Histomorphometric analysis of digitized biopsies.

All histomorphometric analyses were performed using the Olympus cellSens or Leica Aperio ImageScope software for biopsies digitized on Olympus VS120 or Leica SCN400 brightfield scanners, respectively.

#### Duodenal histomorphometry.

**Figure 1.**
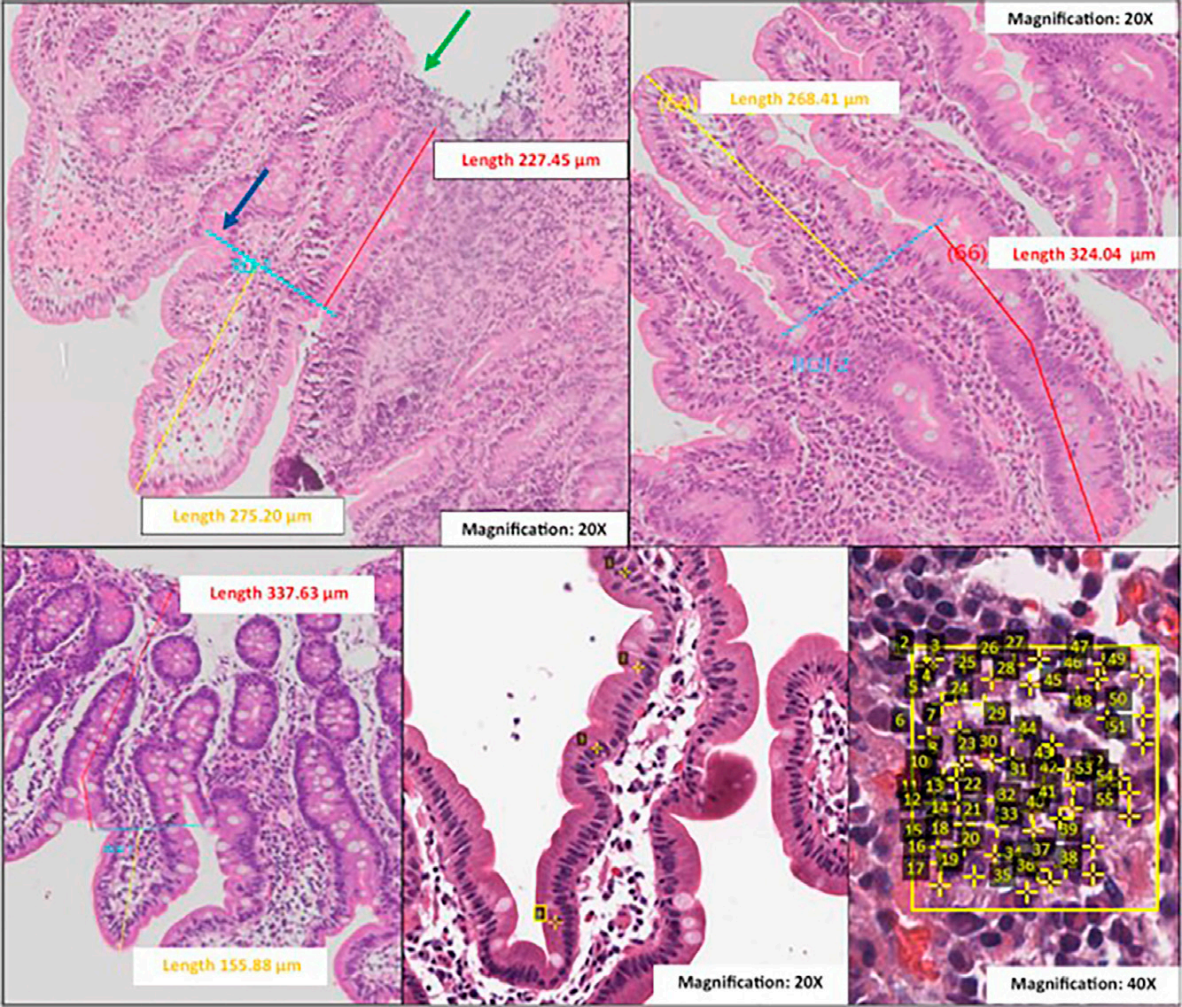
Histomorphometry methods. (**A**) Villus length and crypt depth morphometric measurements. Green arrow: visualization of the end of the mucosa or the beginning of the submucosa enabled assessment of the depth of the mucosa. Blue arrow: villus “shoulder” at the crypt-villus junction. The light blue line represents the end of the first region of interest. (**B**) Crypt depth measurement takes into account the curvature of the crypt. (**C**) Crypt depth measurement (red line) from the crypt-villus junction to the start of the submucosa or the end of the mucosa, where the complete crypt was not visible. This was done to reduce the risk of underestimating crypts in the absence of suitable orientation. (**D**) Quantification of lymphocytes per 100 epithelial cells (analogous method was used for neutrophils and goblet cells per 100 epithelial cells). (**E**) Quantification of mononuclear inflammatory cells/2,500 µm^2^ of lamina propria (analogous method was used for neutrophils and eosinophils/2,500 µm^2^ of lamina propria).

Villus and crypt measurements of the duodenal biopsies were conducted on well-oriented villus-crypt units that were representative of each biopsy image and for which the assessment of the depth of the mucosa was possible. Distances from the villus tip to the crypt-villus junction and then the crypt base were measured in micrometers (µm), as shown in Figure 1A. A horizontal line was placed at the crypt-villus junction (identified via shouldering; a villus “shoulder” was defined as the region where a scaffold was present, which marked the beginning of a crypt) as a point of reference to aid in accurately measuring villus length and crypt depth (Figure 1A). In cases where the villus was curved or bent, the measurements took the curvature into account as supported by previously published literature^19^ (Figure 1B). For crypt depth measurement in a subset where the muscularis mucosa or the beginning of the submucosa was visible without the complete crypts being evident, we extended the measurement from the crypt-villus junction to the muscularis mucosa or the start of the submucosa (Figure 1C). This was done to reduce the risk of underestimating crypts in the absence of suitable orientation, as reported elsewhere.^20^^,^^21^ Those sections without a visible submucosa for which an assessment of the depth of mucosa could not be evaluated were excluded.

To quantify cells in the surface epithelium, IELs, goblet cells, and neutrophils were measured per 100 epithelial cells; surface epithelial cells within the villi only (and not the crypts) were selected. Areas of damaged surface epithelium with breaks and nuclear crowding were avoided unless an increase in nuclear counts was present throughout the biopsy image. Those cells situated at the basal membrane of the surface epithelium were disregarded as it was difficult to distinguish whether they were truly within the surface epithelium or the lamina propria (Figure 1D).

For the quantification of cells in the lamina propria, mononuclear inflammatory cells (MICs), neutrophils, and eosinophils were quantified per 2,500 µm^2^ of lamina propria in each duodenal biopsy.^22^ Overly or deficiently dense cellular areas were disregarded to avoid over-representation of cell counts when counting MICs, neutrophils, and eosinophils (Figure 1E).

#### Rectal histomorphometry.

For the quantification of cells within crypt cross sections, a representative 100,000-µm^2^ region was selected, and the number of crypts within this region was noted.^23^ If greater than 50% of the crypt was within the specified region, then the entire crypt was counted. Overly or deficiently dense crypt epithelium was disregarded to avoid over-representing cell counts. The number of lymphocytes, eosinophils, and neutrophils within the selected crypts was counted. Inflammatory cells within the rectal biopsy lamina propria were quantified using methods analogous to those used for duodenal biopsies.

### EED and celiac disease severity scoring.

Gastrointestinal pathologists used the histologic scoring index developed by the Environmental Enteric Dysfunction Biopsy Initiative (EEDBI) consortium to score the prospective EED (Z. A. and R. I.) and AKU archival biopsies (C. M.).^9^ This scoring system comprises 11 variables with a total of 37 points (see Supplemental Appendix 1 for a detailed EED histologic scoring index). Celiac disease severity was assessed using the Marsh–Oberhüber classification (modified Marsh score), which involves evaluating duodenal villus architecture and intraepithelial lymphocytosis.^24^ In the case where more than one biopsy fragment was present per image, each fragment was separately assessed using the Marsh–Oberhüber classification (see Supplemental Appendix 2 for a detailed illustration of the modified Marsh score classification).

### Data analysis of morphometric measurements.

Regarding data analysis, the morphometric measurements are presented in dot plots with the median (interquartile range [IQR]) marked. The Kruskal-Wallis test was performed to evaluate statistical differences between the groups using GraphPad Prism version 9.04 (GraphPad Software, La Jolla, CA; www.graphpad.com). Multiple comparisons were corrected using Dunn’s multiple comparisons test. Spearman correlation was applied to assess the association between histopathologic features and morphometric measurements. A Mann-Whitney test was used to compare the celiac (Pakistan) and celiac (U.S.) cohorts. A *P* value < 0.05 was considered statistically significant.

### Machine learning–based biopsy image analysis model.

**Figure 2.**
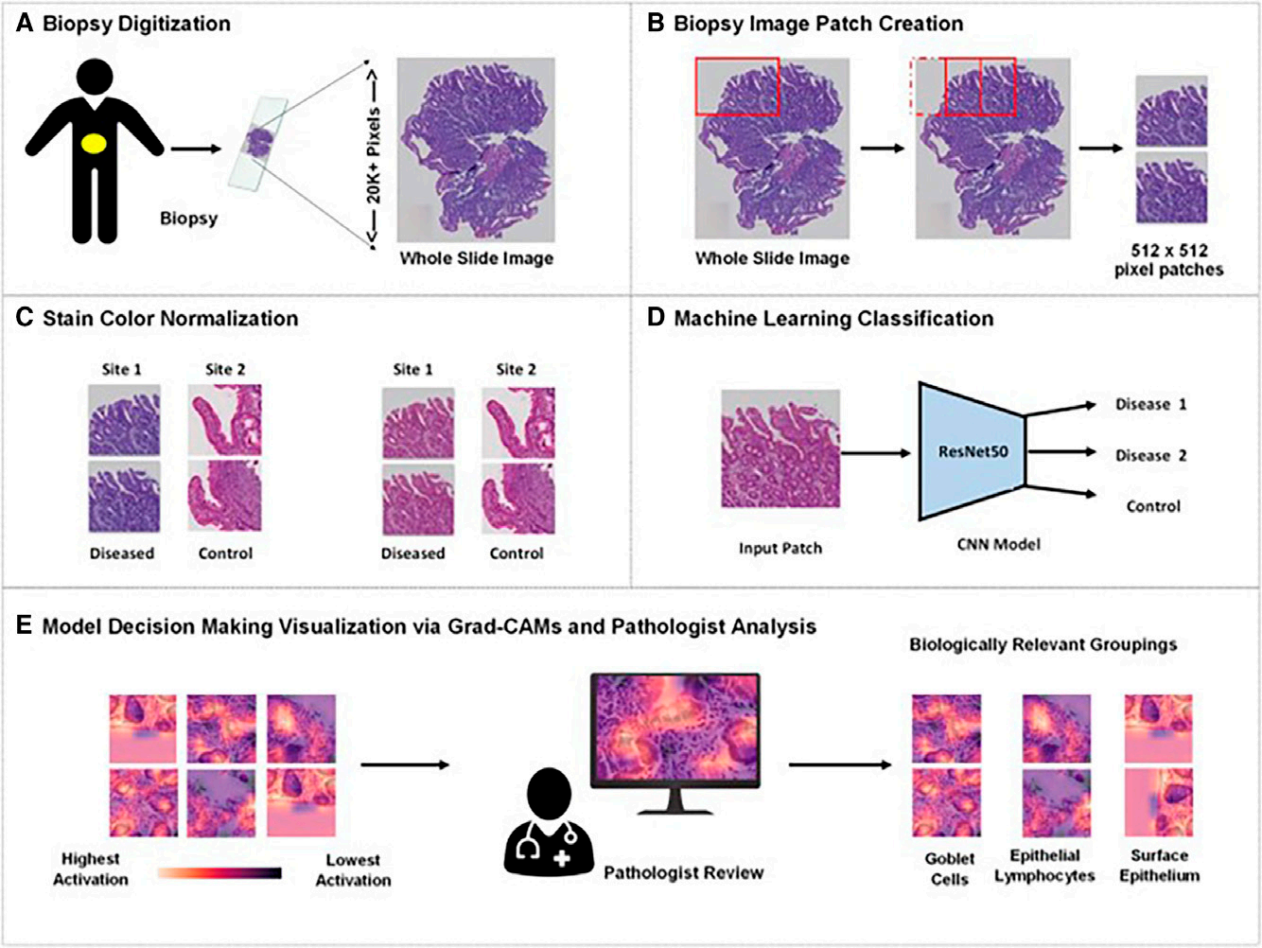
Machine learning biopsy image analysis platform. Methods for the process of data preparation and training. (**A**) Digitization of biopsies as whole slide images (WSIs). Archival H&E-stained biopsies were digitized, resulting in WSIs of more than 20,000 by 20,000 pixels. (**B** and **C**) Biopsy image patch creation and stain color normalization. The WSI was cropped into 512 × 512–pixel patches (rectal tissue) (as shown by the red boxes), which underwent preprocessing for data augmentation and to normalize stain color across biopsies. (**D**) Machine learning classification. The patches were used to train a ResNet50 model to differentiate between diseased and controls. (**E**) Model decision-making visualization and pathologist review. Grad-CAMs were used to visualize the model’s decision-making, which were then reviewed by a trained pathologist who grouped them based on biologically relevant features (goblet cells, epithelial lymphocytes, surface epithelium, etc.). Grad-CAM = Gradient-weighted Class Activation Mappings; H&E = hematoxylin-eosin. CNN = convolutional neural network.

Methods for the process of data preparation and training the machine learning image analysis model are summarized in Figure 2. High-resolution whole slide images (WSIs) that measure more than 20,000 × 20,000 pixels were divided into patches measuring 512 × 512 pixels for analysis within the computational constraints of the model. Each biopsy WSI generated an average of 290 image patches. Details of biopsy image patch creation are explained in Supplemental Appendix 3.

To eliminate bias due to color differences, stain color normalization used the structure-preserving method described by Vahadane et al.^25^ (details in Supplemental Appendix 4). Three independent pathologists (Z. A., R. I., and L. C.) completed a blind review of the color-normalized biopsy images from different sites to assess the structure-preserving ability of the method, details of which have been previously published.^15^

The patches were classified using a pretrained ResNet50 machine learning architecture,^12^ as shown in Figure 2. This machine learning–based classification model was used to evaluate both duodenal and rectal biopsy tissues to help identify patterns of distinguishing morphologic features characterizing duodenal and rectal inflammation.^12^ Details of the model architecture are described in Supplemental Appendix 5. The models were trained on biopsies from patients with EED, celiac disease, and control cases and were then tested on a separate cohort of celiac disease and chronic duodenitis for predictive accuracy. For the classification of rectal biopsies, models were trained using rectal biopsies from children with EED, diseased, and control rectal tissue to predict a separate set of EED, diseased, and control patients.

Gradient-weighted Class Activation Mappings (Grad-CAMs) were used to visualize the regions of interest used by the model for decision-making.^26^ Grad-CAMs were reviewed by a gastrointestinal pathologist (Shyam Raghavan) and a pediatric gastroenterologist (Sana Syed) to enable corroboration of model results with the EED and Marsh histologic scoring indices to assess whether features highlighted by the model as being of high significance in machine learning–based decision-making could be biologically explained (see Supplemental Appendix 6 for details).

## RESULTS

The background characteristics of the study cohorts are summarized in Tables 1 and 2. Further details regarding retrieval of archival biopsies are mentioned in Supplemental Appendix 7.

**Table 1:** Distribution of cases with duodenal tissue hematoxylin-eosin–stained histology slides

Variables	Pakistan				United States	
	EED	Celiac	Chronic duodenitis	Undiagnosed	Celiac	Controls
*n*	63	18	17	7	63	61
Median (IQR) age, months	20.7 (15.3, 22.2)	42 (25.5, 48)	48 (42, 60)	48 (36, 60)	130 (92.5, 175.5)	25 (17, 41)
Male gender, %	69.8	22.2	52.9	42.9	46.0	54.1
LAZ/HAZ, median (IQR)	−2.04 (−2.83, −1.30)	−1.82 (−2.76, −0.84)	−1.45 (−1.75, −0.14)	−0.68 (−1.63, −0.04)	−0.34 (−0.84, 0.65)	−0.22 (−1.25, 0.49)

EED = environmental enteric dysfunction; IQR = interquartile range; LAZ/HAZ = length/height-for-age *Z* score. Missing length/height for duodenal biopsies: Pakistani celiac (*N* = 4), chronic duodenitis (*N* = 2), undiagnosed (*N* = 1).

**Table 2:** Distribution of cases with rectal tissue hematoxylin-eosin–stained histology slides

Variables	Pakistan	United States		
	EED	Controls	Ulcerative proctitis	Cryptitis
*n*	14	9	8	4
Median (IQR) age, months	21.5 (20.5, 22.7)	165 (50, 196.8)	141 (111.8, 193.8)	72 (67, 88.5)
Male gender, %	71.4	33.3	25.0	25.0
LAZ/HAZ, median (IQR)	−1.89 (−2.56, −0.98)	0.02 (−0.57, 0.54)^4^	−0.02 (−0.12, 0.19)	0.49 (0.14, 1.02)

EED = environmental enteric dysfunction; IQR = interquartile range; LAZ/HAZ = length/height-for-age *Z* score. Missing length/height for rectal biopsies: cryptitis (*N* = 1), controls (*N* = 1).

### Morphometric features of EED and celiac disease.

**Figure 3.**
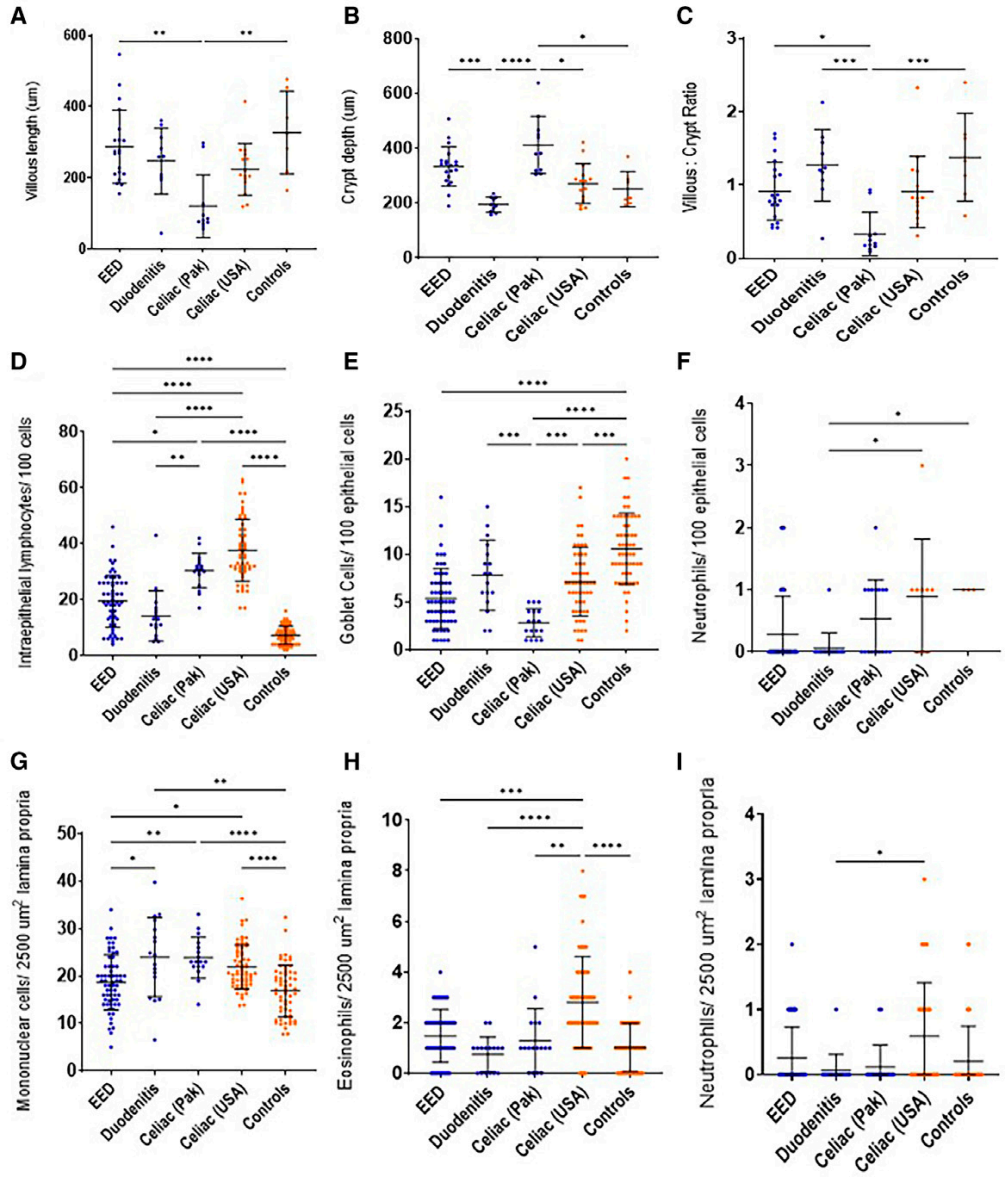
Duodenal histomorphometry. Comparison of villus architecture measurements (**A**–**C**), cells per 100 epithelial cells (**D**–**F**), and in the lamina propria per 2,500 μm^2^ (**G**–**I**). Cohorts from Pakistan are shown as blue dots, whereas those from the United States are in orange. The Kruskal-Wallis test was applied to assess for *P* value between various groups, indicated as black bars where significant. *P < 0.05, **P < 0.005, ***P < 0.0005, ****P < 0.0001. (**A**–**C**) Sample size: EED = 20, duodenitis = 10, celiac (Pak) = 11, celiac (United States) = 15, controls = 7. (**D** and **E**) Sample size: EED = 60, duodenitis = 17, celiac (Pak) = 17, celiac (United States) = 56, controls = 58. (**F** and **I**) Sample size: EED = 63, duodenitis = 17, celiac (Pak) = 17, celiac (United States) = 39, controls = 34. (**G** and **H**) Sample size: EED = 63, duodenitis = 16, celiac (Pak) = 17, celiac (United States) = 62, controls = 42. EED = environmental enteric dysfunction; Pak = Pakistan. The error bars represent median with interquartile range.

The villus blunting on the duodenal sections was most prominent in celiac disease, with a median (IQR) length in controls of 324 (218, 454) µm. Pakistani cases of celiac disease demonstrated even shorter lengths than U.S. celiac cases, with medians (IQR) of 81 (73, 127) µm and 209 (188, 266) µm, respectively; this was also reflected in the low villus-to-crypt ratio, which indicates damage (villus) and regenerative (crypt) responses (Figure 3A–C). Chronic duodenitis had minimal impact on the villus architecture. The villus length of cases with EED was nonsignificantly lower than that of controls, with a median (IQR) of 273 (211, 330) µm. Concerning crypt depth, Pakistani celiac cases reported maximum depth at a median of 382 (319, 466) µm, followed by EED cases with a median (IQR) of 339 (286, 354) µm.

Goblet cell depletion and increased IELs were common features of both EED and celiac disease. The median goblet cell count in controls was 10 cells per 100 epithelial cells, whereas a significantly lower count was observed in EED cases (5 [3, 7] cells) (Figure 3E). Similar patterns of depletion were seen in celiac disease, with a more pronounced reduction in cases from Pakistan (3 [1.5, 4] cells) compared with their U.S. counterpart (7 [4, 10] cells), suggesting that goblet cell depletion is an important feature of regional variations.

Regarding inflammatory cells in the surface epithelium and lamina propria, an increase in IELs is a known feature of celiac disease and was seen to be heightened in disease cases from Pakistan and the United States. Environmental enteric dysfunction samples had significantly higher IELs per 100 epithelial cells count than controls, with medians (IQR) of 19 (12, 26) and 7 (4, 9), respectively, although both groups had lower IEL counts than those observed in celiac cases (Figure 3D and F). Mononuclear inflammatory cells were prominent in duodenitis cases, with a median (IQR) of 24 (18, 31) per 2,500 μm^2^, followed by celiac disease (23 [22, 27]) per 2,500 μm^2^ (Figure 3G). Cases with EED reported a nonsignificant increase in MICs compared with controls (Figure 3H). Neutrophils were mostly seen in the lamina propria of cases with celiac disease (Figure 3I). A comparison of all groups that include seven undiagnosed Pakistani samples is summarized in Supplemental Figure 2.

### Evaluation of duodenal sections per EED consortium scoring and Marsh scoring.

**Figure 4.**
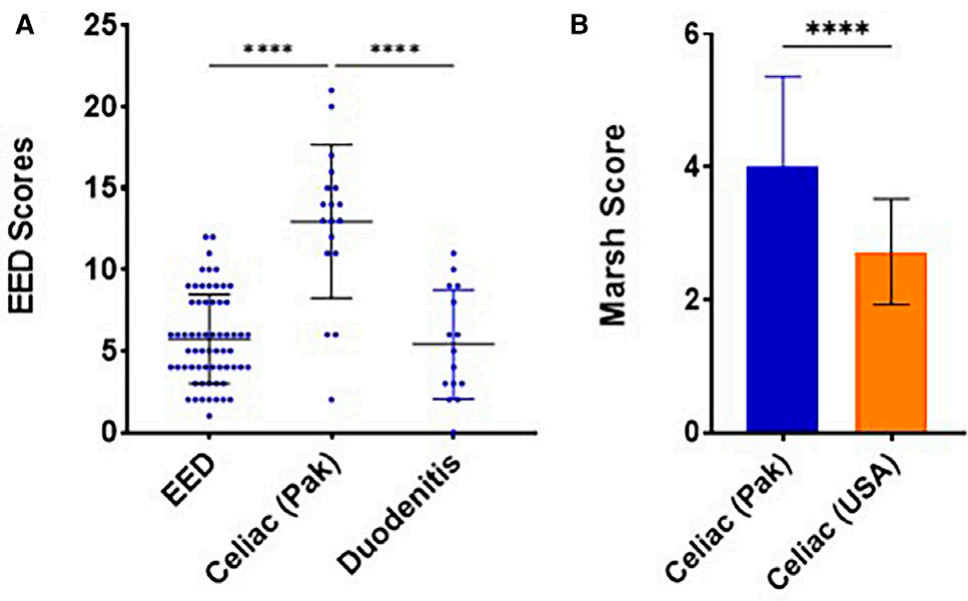
Comparison of scores assessed on duodenal sections. (**A**) Environmental enteric dysfunction (EED) biopsy initiative consortium scoring out of 37. The sample size of each group was EED = 63, celiac (Pakistan) = 18, and duodenitis = 15. (**B**) Marsh scores are compared between celiac cases from two geographical locations, Pakistan and the United States. ****P < 0.0001. The error bars represent median with interquartile range. Pak = Pakistan.

The total severity scores of cases from Pakistan with EED using the EEDBI consortium scoring scheme have already been published.^18^ Upon assessment of celiac disease slides using the same scoring system, morphologic changes in duodenal histology were significantly more pronounced than EED cases, with average scores of 5 (4, 8) and 14 (11, 15), respectively (Figure 4A). Interestingly, in the cohort of undiagnosed cases, we observed similar severity scores as in the cohort with EED, suggesting the presence of some abnormalities not highlighted on initial histopathologic evaluation (Supplemental Figure 2J).

Celiac disease cases from Pakistan had overall higher Marsh scores than celiac cases from the United States (Figure 4B). As previously highlighted, compromised villus architecture and goblet cell depletion were markedly more drastic in Pakistani samples, whereas eosinophils were more commonly seen in the lamina propria of celiac cases from the United States. However, intraepithelial inflammation and mononuclear infiltration of connective tissue were observed to be similar in the two groups (Figure 3).

### Mononuclear inflammatory cell infiltration as a feature of EED on rectal histomorphometry.

**Figure 5.**
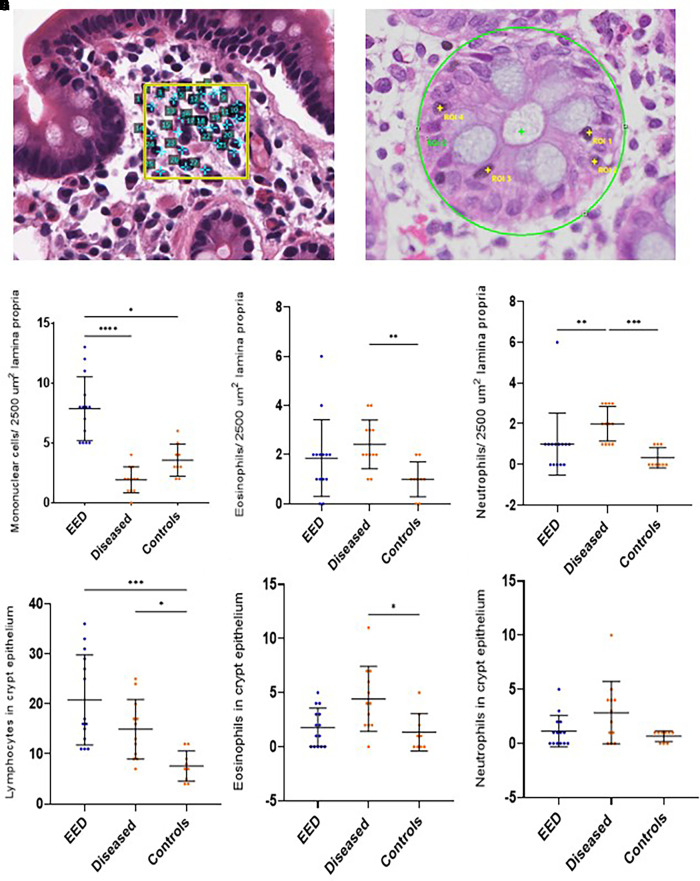
Rectal histomorphometry. (**A** and **B**) Rectal morphometry shown on photomicrographs taken at ×40 magnification. (**C**–**H**) Quantification of mononuclear cells, eosinophils, and neutrophils per 2,500 μm^2^ of lamina propria (**C**–**E**) and crypt cross sections within a region of 100,000 μm^2^ (**F**–**H**). The sample sizes of each group were EED = 14, diseased = 12, and controls = 9. The Kruskal-Wallis test was applied to assess the *P* value between various groups. The error bars represent median with interquartile ranges. *P < 0.05, **P < 0.005, ***P < 0.0005, ****P < 0.0001. EED = environmental enteric dysfunction. ROI = region of interest.

The presence of increased MICs and IELs in the crypts was the most striking features of EED rectal morphometry (Figure 5A and D), whereas the presence of eosinophils and neutrophils was more pronounced in the diseased rectal tissue (Figure 5B, C, E, and F). Inflammatory cells were rarely seen on normal tissue.

On rectal morphometry, increased mononuclear cells in the lamina propria and the presence of lymphocytes in the epithelial lining of the crypts were found to be distinguishing features of rectal biopsies collected from EED cases. Neutrophil and eosinophil counts in the lamina propria and crypt epithelium were more commonly seen in biopsies collected from ulcerative proctitis or cryptitis (Figure 5G and H). However, these cells were also seen in EED sections.

### Association of duodenal and rectal morphometry with EED scoring.

**Figure 6.**
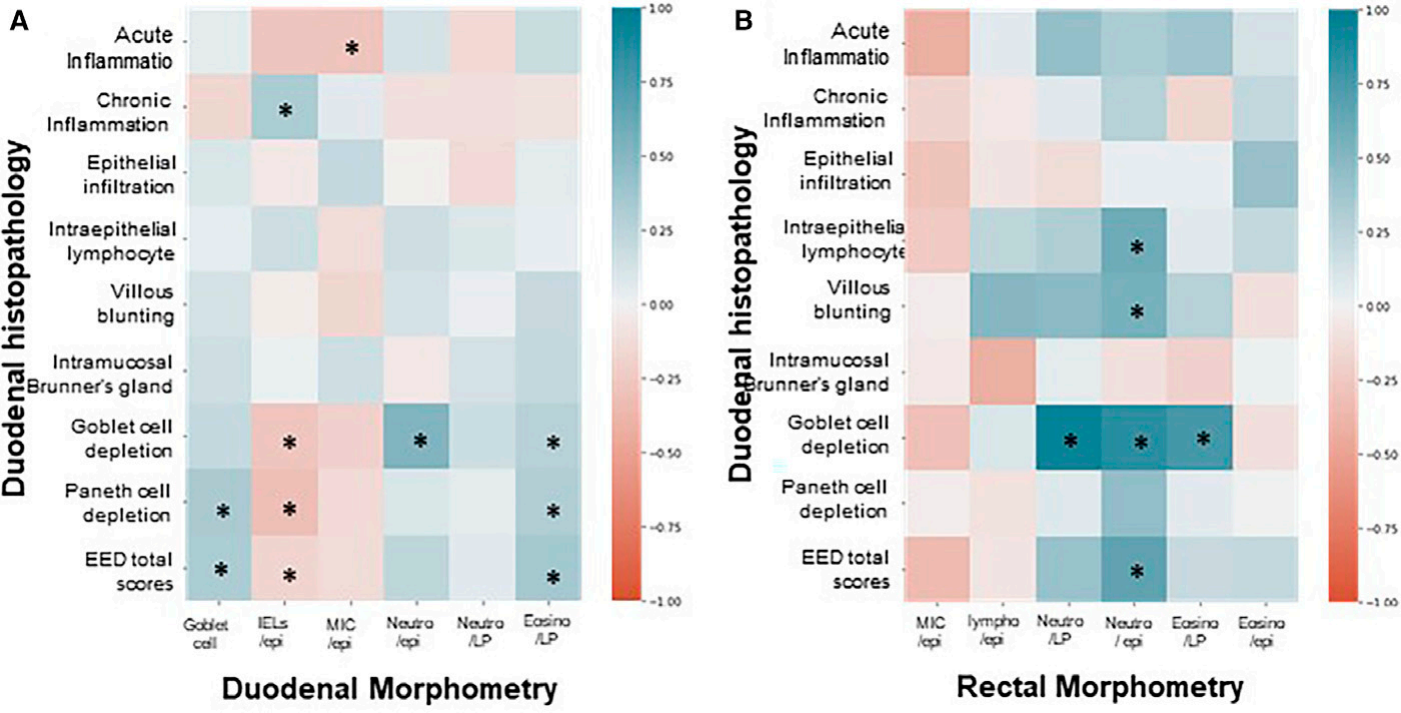
Correlation heatmap between EED histopathology scoring features and morphometry. (**A**) Comparison of duodenal features where histology features are lined on the *y* axis, and morphometric measurements are on the *x* axis. (**B**) Comparison of rectal features with histology features on the *y* axis, and rectal morphometric measurements are on the *x* axis. Positive or negative associations with significant associations (*P* < 0.05) are marked with *. EED = environmental enteric dysfunction; Eosino/LP = eosinophils in lamina propria; IEL/epi = intraepithelial lymphocytes in epithelial cells; MIC/epi = mononuclear inflammatory cells in epithelial cells or in lamina propria; Neutro/epi = neutrophils in epithelial cells; Neutro/LP = neutrophils in lamina propria.

Intraepithelial lymphocytes on duodenal morphometry were associated with chronic inflammation and IELs on histopathology but not acute inflammation (Figure 6A). On morphometry, eosinophil counts in the lamina propria showed a significant association with the depletion of goblet cells and Paneth cells, along with total EED scores. The presence of neutrophils in the epithelium and lamina propria was associated with some histologic features, yet it did not reach statistical significance. Regarding rectal morphometry, the presence of neutrophils within the epithelial lining of the crypt reported the most significant associations with histology features of the duodenum (Figure 6B).

### Machine learning models for biopsies and Grad-CAMs.

**Figure 7.**
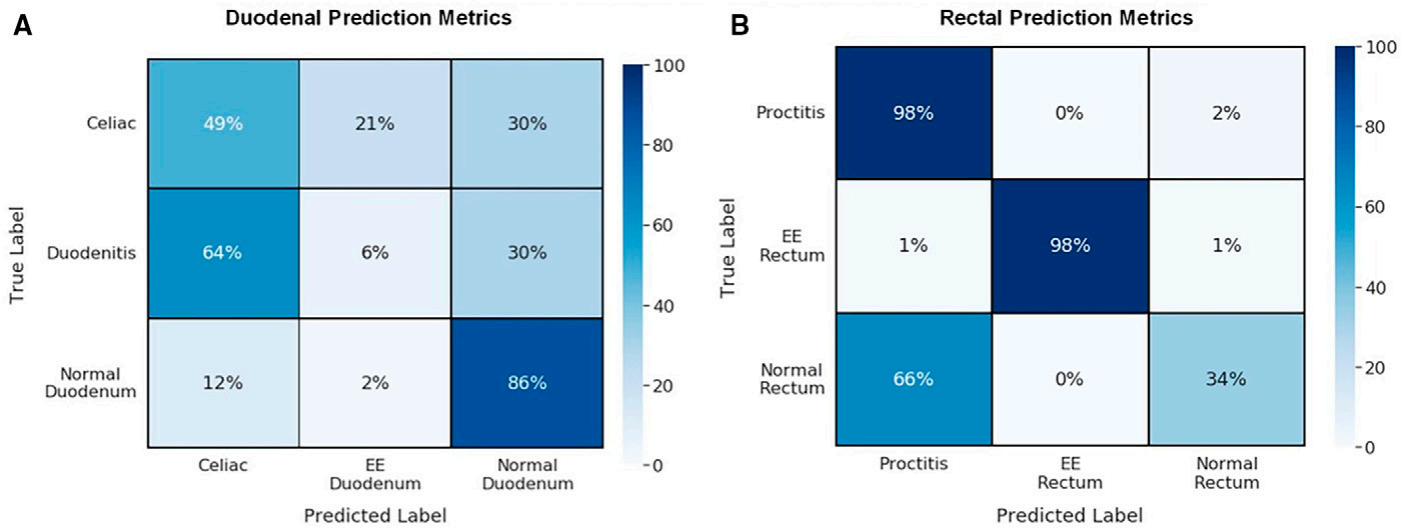
Confusion matrices showing classification results from the machine learning-based image analysis models. Classification results of the duodenal (**A**) and rectal (**B**) machine learning biopsy image analysis model shown as confusion matrices. The numbers within the matrices represent normalized data in the form of percentages, and darker colors indicate higher percentages. EE = environmental enteropathy.

**Figure 8.**
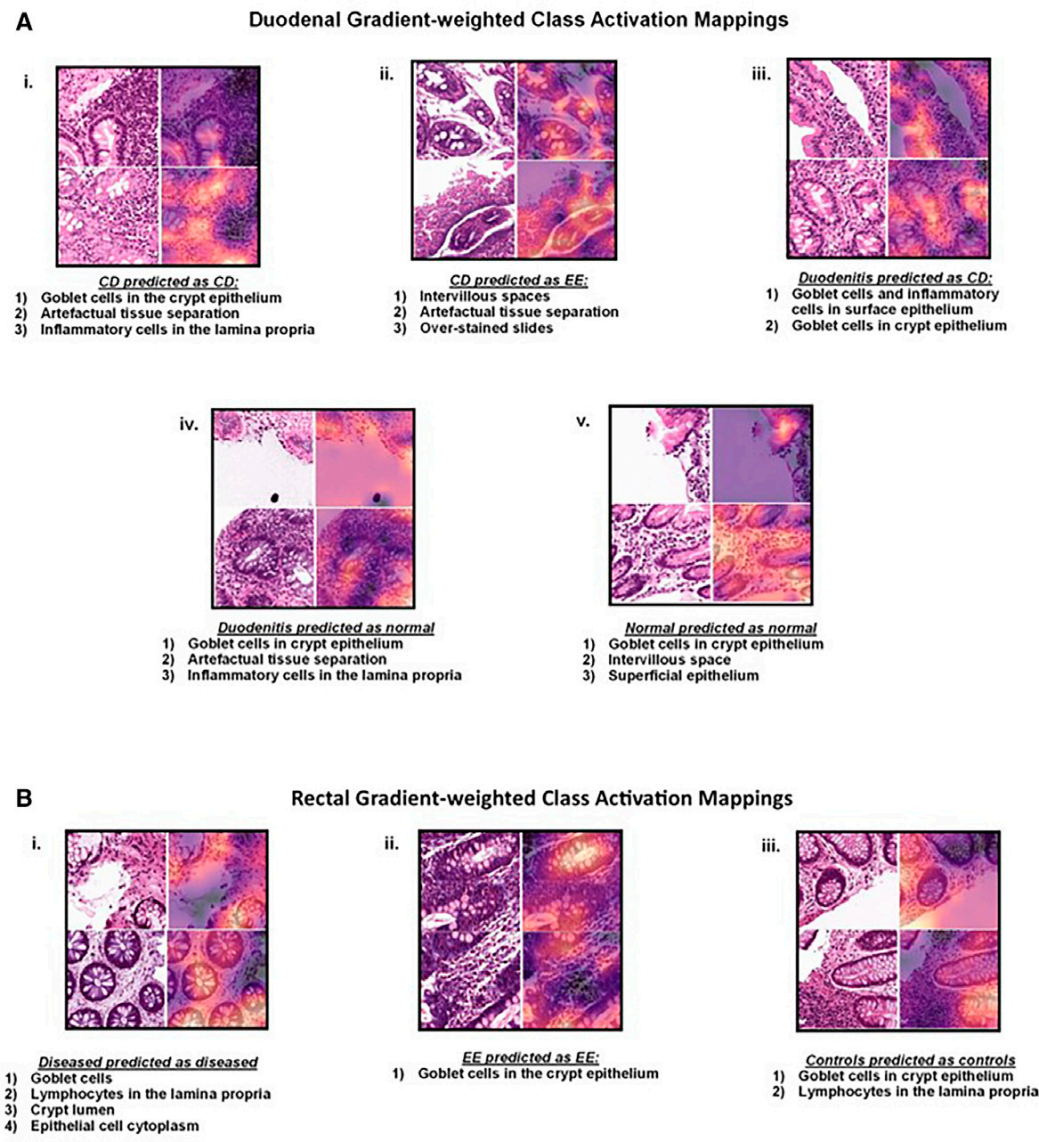
Gradient-weighted Class Activation Mappings (Grad-CAMs) for patches trained via ResNet50 are shown. (**A**) Duodenum. Salient features of biopsies (i.) with celiac disease that were predicted as celiac disease; (ii.) with celiac disease that were classified as environmental enteropathy; (iii.) with duodenitis that were predicted as celiac disease; (iv.) with duodenitis that were predicted as having no disease; (v.) with no disease that were predicted correctly. (**B**) Rectum Salient features of biopsies (i.) with disease that were predicted as disease; (ii.) with environmental enteropathy that were correctly classified; (iii.) from controls that were predicted as controls. CD = celiac disease; EE = environmental enteropathy.

Regarding duodenal tissue, the ResNet50 machine learning model exhibited an accuracy of 49%, 21%, and 30% for predicting celiac disease biopsies as diseased, EED, and normal, respectively (Figure 7A). The model also exhibited 64%, 6%, and 30% accuracy for predicting chronic duodenitis biopsies as celiac disease, EED, and normal. Finally, it exhibited 12%, 2%, and 86% accuracy for predicting chronic duodenitis biopsies as celiac disease, EED and normal. Grad-CAMs are shown in Figure 8A. The model was performed on rectal tissue. The model predicted diseased rectal biopsies with an accuracy of 98% and EED with an accuracy of 97% (Figure 7B). The normal biopsies overlapped with diseased (66%) with a 33% prediction accuracy for normal. Grad-CAMs for rectal classification are shown in Figure 8B.

## DISCUSSION

This study aimed to characterize duodenal and rectal tissues among children with EED using histomorphometry. In addition to duodenal features, rectal inflammation previously reported in the last century has been observed in a subset of children who underwent colonoscopy in addition to upper endoscopy, highlighting the potential involvement of the large intestine in EED. Compared with celiac disease patients, duodenal tissue in patients with EED showed milder histopathologic changes. Archival samples with a lack of findings on histopathology were evaluated to serve as local controls. However, pathologic findings were seen on morphometry and EED scoring and were excluded from the analysis.

Widespread variations in morphometric measurements were observed across EED samples, suggesting a spectrum of severity that supports previous histopathology findings.^13^ Goblet cell depletion and IELs were marked in EED samples compared with controls, whereas severely altered villus architecture was a feature of celiac disease. Inflammatory cells in the lamina propria were commonly observed in both duodenitis and EED. Goblet cells secrete mucin that functions during primary defense against pathogens and downregulation in mucin production is commonly seen in enteropathies and have been previously observed in patients with EED.^27^ As EED is often an asymptomatic condition, milder inflammation seemingly contributes to compromised gut absorption, leading to growth restriction.^1^ These findings are supported by a study of otherwise healthy Gambian children who demonstrated similar duodenal morphometry to that of severely malnourished children.^28^ However, the lack of in-country controls with no histopathologic abnormalities is a limitation of this study. Future studies may provide improved morphometric comparisons through the inclusion of duodenal samples collected from children with foreign body inhalation (without any pathological findings) to serve as in-country controls.

When celiac cases from the two countries were compared, goblet cell depletion was more pronounced in Pakistani cases, whereas eosinophilic infiltration in U.S. cases suggest regional differences in disease presentation. These differences were further confirmed by Marsh score differences in the two populations. This suggests a geographic predisposition toward developing a more pronounced enteric inflammatory response and, subsequently, more histologically abnormal enteropathies in the resource-limited setting in comparison to the higher-income setting. Therefore, morphometric comparisons between healthy tissues from developed countries and LMICs to determine geographic differences, in consortia that include pathologists from both settings, are worth exploring.

In machine learning–driven tissue analyses, Grad-CAM saliency maps for celiac disease and chronic duodenitis focused on goblet cells, which are known to decrease in density with intestinal inflammation.^9^ Our previous models also focused on goblet cells in the presence of duodenal inflammation.^11^^,^^12^ We could not assess goblet cell density, as Grad-CAMs provide a qualitative rather than a quantitative assessment. For biopsies from patients with celiac disease that were classified as EED, there was a focus on intervillus space and artefactual tissue separation. Our previously published model also focused on areas of white slit-like spaces representing the artefactual separation of tissue within the lamina propria as a feature of EED.^12^ Based on our machine learning analysis, children in resource-limited countries diagnosed with EED or chronic duodenitis may potentially have some overlapping components of underlying celiac disease. Previously published studies indicate an underestimation of the prevalence of even well-established enteropathies, such as celiac disease, in South Asia as a result of lack of resources for diagnosis, further supporting our findings.^29^^,^^30^

Our rectal histomorphometry insights point toward more significant colonic involvement in EED than was previously understood. Mathan et al.^15^ demonstrated increased inflammatory cells in the rectal mucosa of healthy Indian volunteers, resembling the nonspecific inflammatory response in the small intestine associated with EED (known then as tropical enteropathy) and hypothesized the existence of tropical colonopathy.^15^ In our study, rectal biopsies were collected only from children with fecal bleeding and demonstrated a significantly higher number of inflammatory cells in the mucosa. This suggests that EED may not be limited to the small intestine; thus, improving colonic health may be added to the strategies for treatment. In children with short bowel syndrome and subsequent malabsorption, the large intestine acts as a salvage organ for increasing caloric intake via the absorption of carbohydrates.^31^ Therefore, in small intestinal malabsorption syndromes such as EED, compromised ability of absorption through the large intestine may hinder the body’s efforts to compensate for absorption through colonic tissue. These findings must be further explored in rectal tissue collected from EED cases, with careful consideration to study colopathy in patients both with and without rectal bleeding to rule out other colonopathies.

Rectal tissue Grad-CAM saliency map analyses highlighted areas of goblet cells, lymphocytes in the lamina propria, and surface epithelial cells. These findings are similar to lymphocytic colitis, distinguished from celiac sprue by increased lamina propria cellularity, surface epithelial abnormalities, and fewer IELs.^32^ Even though our machine learning–based analysis model for rectal biopsy analysis demonstrated high classification accuracies for predicting both diseased (98%) and EED (98%) colon, our small sample size precludes us from drawing meaningful interpretations at this time. However, similar to our model for duodenal analysis, more data will strengthen our analyses and enable us to predict disease characteristics that will aid in elucidating tissue features of EED-related colonopathy.

One of the major strengths of our study is the expansion of our understanding of colonopathy among children with EED. We compared duodenal tissue to enteropathies other than celiac disease. Further, we demonstrated the ability of machine learning–based image analysis to identify key distinguishing features in both diseased and normal tissues. Grad-CAMs may increase physician confidence in machine learning–based decision-making and pave the way for discerning disease-specific histopathologic markers.

However, our study does have several limitations. First, the sample size for the rectal analysis was limited, and we acknowledge that more data will be required before meaningful conclusions can be drawn. Inflammation in the lamina propria was quantified by mononuclear cells that include lymphocytes, plasma cells, dendritic cells, and macrophages; this is a limitation of H&E–stained biopsies. Immunohistochemistry can be used to better characterize cells in the lamina propria. Third, histomorphometry can be subject to observer bias, and interobserver analyses were beyond our scope. Orientation of the tissue is also an important feature while performing morphometry, and as a result, we were able to evaluate villus architecture in only a subset of duodenal biopsies. Lastly, demographic data for archival samples were limited to gender and age (with a diverse range of ages up to 18 years, as gut tissue does not exhibit age-related differences).

In conclusion, EED comprises a spectrum of inflammation in the duodenum that has been observed in Pakistani children in comparison to their U.S. counterparts and needs further exploration in other settings. In addition, the involvement of the rectal mucosa merits further investigation, especially in the context of loss of compensatory absorption from the colon.

## Supplemental files


Supplemental materials
