# Supplementary material for: Quantitative Morphometry and Machine Learning Model to Explore Duodenal and Rectal Mucosal Tissue of Children with Environmental Enteric Dysfunction
Source: Am J Trop Med Hyg. 2023 Mar 13;108(4):672–83. doi: 10.4269/ajtmh.22-0063 (PMC10077000; doi:10.4269/ajtmh.22-0063)
Supplement: Supplementary file 1 [file tpmd220063.SD1.pdf]

Supplemental Methods:

Appendix S1. Supplemental Methods – Environmental Enteropathy (EE) Scoring

EED scoring [1] included assessment of the following features: acute inflammation (score 0-3), eosinophil infiltration (score 0-3), chronic inflammation in the lamina propria (score 0-3), intra-epithelial lymphocytes (score 0-4), villus architecture (score 0-4), intramucosal brunner glands (score 0-3), foveolar cell metaplasia (score 0-3), goblet cell density (score 0-3), Paneth cell density (score 0-3), enterocyte injury (score 0-3), and epithelial detachment (score 0-4). Detailed description of the scoring of each feature is shown in the table below:

Feature	Grade	Description
Acute inflammation	0	No PMNs observed, or only PMNs in lamina propria with no infiltration of epithelium by PMNs (cryptitis, villitis)
	1	1-2 foci of epithelial PMN infiltration or crypt microabscesses
	2	> 2 foci of epithelial PMN infiltration or crypt microabscesses but <50% of mucosa involved
	3	≥ 50% of mucosa involved by epithelial PMN infiltration
Eosinophil infiltration	0	No increase in eosinophils (highly scattered in lamina propria, no intravillus or intercryptal space with >5 eosinophils)
	1	Increased eosinophils (intravillus or intercryptal space with >5 eosinophils) involving < 50% of mucosa, with no eosinophilic crypt microabcesses
	2	Increased eosinophils (intravillus or intercryptal space with >5 eosinophils) involving > 50% of mucosa, or up to 1 focus of eosinophilic epithelial infiltration or crypt microabcesses per mucosal fragment
	3	>2 foci of eosinophilic epithelial infiltration or crypt microabcesses in any mucosal fragment
Chronic inflammation-lamina propria	0	No qualitative increase in mononuclear inflammatory cells (MIC) in lamina propria. Majority of villus bases contain ≤3 MIC across, on average.
	1	Increased MIC, based on villus base displaying 3-5 MIC across, on average.
	2	Increased MIC, based on villus base displaying 6-10 MIC across, on average.
	3	Increased MIC, based on villus base displaying >10 lymphocytes on average.
Intra-epithelial lymphocytes	0	No areas observed with epithelial/lymphocyte ratio ≥20%
	1	Lymphocyte/epithelial ratio ≥20%, but <50%, in less than 50% of mucosa
	2	Lymphocyte/epithelial ratio ≥20%, but <50%, in greater than 50% of mucosa
	3	Lymphocyte/epithelial ratio ≥50% in less than 50% of mucosa
	4	Lymphocyte/epithelial ratio ≥50% in greater than 50% of mucosa
Villus architecture	0	Majority of villi are >3 crypt lengths long
	1	Villi are ≤ 3 but > 2 crypt lengths long, in < 50% of mucosa.
	2	Majority of villi are ≤ 2 crypt lengths long, but > 1 crypt length long
	3	Villi absent, or ≤1 crypt length long, in < 50% of mucosa.
	4	Villi absent, or <1 crypt length long, in > 50% of mucosa.
Intramucosal Brunner glands	0	None observed
	1	One or two foci, none involving more than 5 crypt bases
	2	3-5 foci, none involving more than 5 crypt bases
	3	> 5 foci, or any area of intramucosal Brunner glands involving >5 crypt bases
Foveolar cell metaplasia	0	Not observed
	1	1-2 villus tips involved
	2	3-5 villus tips involved
	3	> 5 villus tips involved
Goblet cell density	0	Most villi contain ≥10 goblet cells
	1	Goblet cells <10/ villus, involving < 25% of mucosa
	2	Goblet cells <10/ villus, involving 25-50% of mucosa
	3	Goblet cells <10/ villus, involving >50% of mucosa
Paneth cell density	0	≥5 Paneth cells/ crypt, on average
	1	2-4 Paneth cells/ crypt, on average
	2	<2 Paneth cell/crypt, involving <50% of crypt bases
	3	<2 Paneth cell/crypt, involving >50% of crypt bases

Enterocyte injury	0	Majority of enterocytes (90%) show tall columnar morphology
	1	Enterocytes show low columnar (≤2:1 L:W ratio), cuboidal or flat morphology, in < 50% of mucosa
	2	Enterocytes show low columnar (≤2:1 L:W ratio), cuboidal or flat morphology, in > 50% of mucosa
	3	Any area of mucosal erosion/ulceration
Epithelial detachment	0	Complete coverage of mucosal surface by epithelial cells
	1	Surface epithelium missing or detached from <25% of mucosa
	2	Surface epithelium missing or detached from 25-50% of mucosa
	3	Surface epithelium missing or detached from 51-75% of mucosa
	4	Surface epithelium missing or detached from >75% of mucosa

Appendix S2. Supplemental Methods – Assessment of Celiac Disease Severity via Marsh–Oberhüber Classification for the Duodenum.

Marsh I has been described as normal architecture with more than 30 lymphocytes interspersed between 100 villous surface epithelial cells. Marsh II has shown to include increased intraepithelial lymphocytes (>30) along with crypt hypertrophy; although it is rarely encountered in clinical practice since patients proceed rapidly from Marsh I to IIIa. Marsh III has been further sub-divided into IIIa (partial villous atrophy), Marsh IIIb (subtotal villous atrophy), and Marsh IIIc (complete villous atrophy) [2, 3].

Appendix S3. Supplemental Methods – Biopsy Image Patch Creation.

Whole Slide Images (WSIs) were split into patches of 1000x1000 and 2000x2000 pixels with an overlap in horizontal and vertical axes of 750 and 1000 pixels, respectively. Patches that contained less than 50 percent tissue area were discarded, and the remaining were resized to 256x256 pixels as final input for the model. Each biopsy WSI generated an average of 250 1000x1000 and 40 2000x2000 patches. As there were more biopsy images for CD, EE and control images were up sampled to balance datasets. Machine learning studies have focused on obtaining as much image data as possible without any standard sample size recommendations.

Appendix S4. Supplemental Methods – Biopsy Stain Color Normalization.

Method described by Vahadane et al. [4] preserves structural information and involves empiric selection of a target biopsy image as a reference to normalize coloration across all biopsy images. With this method, the color of all the biopsy images became the same as that of the empirically selected target biopsy image. This was an added layer of mitigating data bias as all our biopsy images were obtained from the same institute but may have varied stain chemical compositions over time.

Appendix S5. Supplemental Methods – Machine Learning Classification Model.

ResNet50 is a widely used ML architecture with 50 layers for image classification requiring identification of microscopic patterns. With the pre-trained ResNet50 ML architecture, different layers capture different information; the layers closer to the input were more likely to have learned more general features, while the later layers identified more abstract features depending on the dataset the model has been trained on. Therefore, the learning rate used while training the initial layers was 1/9th of the rate of the final layers, while the middle layers used a learning rate 1/3rd the rate of the final layers. This ML classification model was used to evaluate both duodenal and rectal biopsy tissue to help identify patterns of distinguishing morphological features characterizing duodenal and rectal inflammation.

Appendix S6. Supplemental Methods – Visualization of Machine Learning Model Decision-making via Gradient-weighted Class Activation Mappings (Grad-CAMs).

Gradient-weighted Class Activation Mappings (Grad-CAMs) generate the localization heatmaps highlighting specific regions of interest [5]. Activation values from an intermediate convolution layer and corresponding gradients as described by Selvaraju et al. [4] to generate these heatmaps.

<u>Supplemental Results</u>

Appendix S7. Study Subjects with Archival Biopsies

Per AKU and Pakistan’s medicolegal policies, informed consent had to be obtained from the patient or parent prior to retrieval of AKU archival biopsies, and we were able to access duodenal biopsies from 44 patients. Chart review of patients belonging to the AKU archival dataset included compiling the following information: date of EGD, histology report, presence of giardia in biopsy tissue, previous histology, tissue transglutaminase antibody titers, clinical information/ history of the patient, including patient’s demographics (age and gender) and anthropometric measures (weight, height, and body mass index).

For UVA archival dataset, 99 celiac disease biopsy reports were identified in children ≤ 18 years who had undergone EGD between 2003 – 2017. 63 patients were selected after excluding multiple biopsies of the same patient and unviability of the biopsy slide (artifacts obscuring duodenal tissue and poorly sectioned biopsies). 243 biopsy reports were identified with normal duodenal histology reported in children who had undergone EGD with biopsy between 2014 – 2017

Supplemental References

1. Liu TC, VanBuskirk K, Ali SA, Kelly MP, Holtz LR, et al. (2020) A novel histological index for evaluation of environmental enteric dysfunction identifies geographic-specific features of enteropathy among children with suboptimal growth. PLOS Neglected Tropical Diseases 14(1): e0007975. https://doi.org/10.1371/journal.pntd.0007975
2. Modified Marsh Classification of histologic findings in celiac disease (Oberhuber). Surgical Pathology Criteria Stanford Medicine.
3. Oberhuber G. Histopathology of celiac disease. Biomedicine & pharmacotherapy 2000;**54**:368-72.
4. Vahadane A, Peng T, Sethi A, Albarqouni S, Wang L, Baust M*, et al.* Structure-preserving color normalization and sparse stain separation for histological images. IEEE transactions on medical imaging 2016;**35**:1962-71.
5. Selvaraju RR, Cogswell M, Das A, Vedantam R, Parikh D, Batra D. Grad-cam: Visual explanations from deep networks via gradient-based localization. Proceedings of the IEEE International Conference on Computer Vision, 2017:618-26.

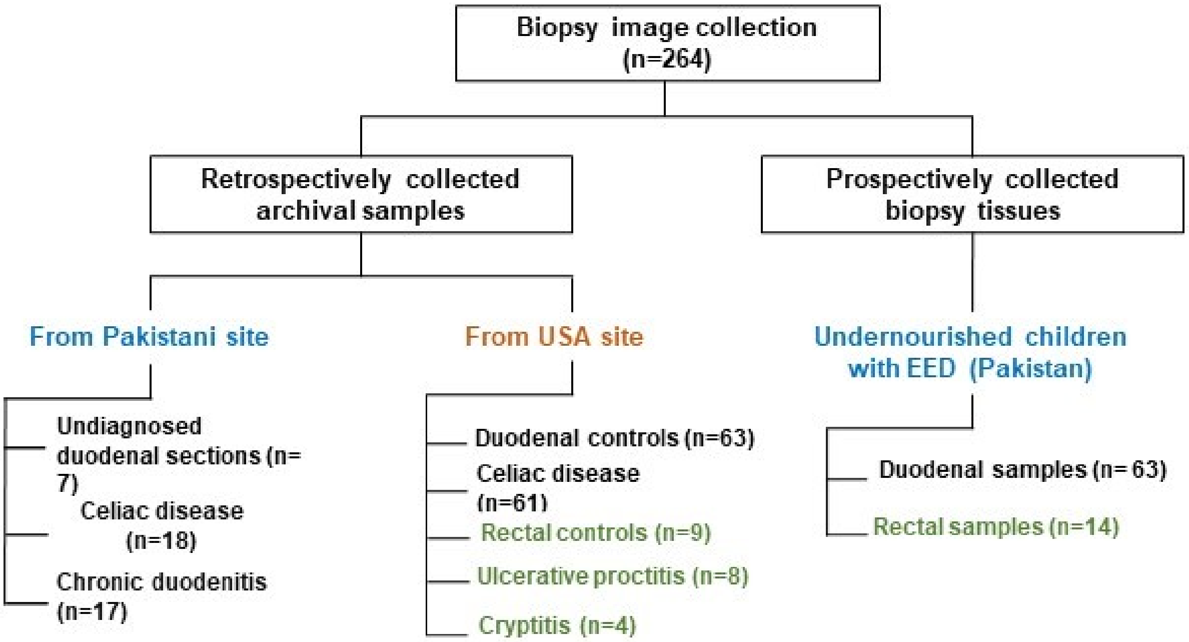
Biopsy image collection
(n=264)
Retrospectively collected archival samples
Prospectively collected biopsy tissues
From Pakistani site
From USA site
Undernourished children with EED (Pakistan)
Undiagnosed duodenal sections (n= 7)
Celiac disease (n=18)
Chronic duodenitis (n=17)
Duodenal controls (n=63)
Celiac disease (n=61)
Rectal controls (n=9)
Ulcerative proctitis (n=8)
Cryptitis (n=4)
Duodenal samples (n= 63)
Rectal samples (n=14)

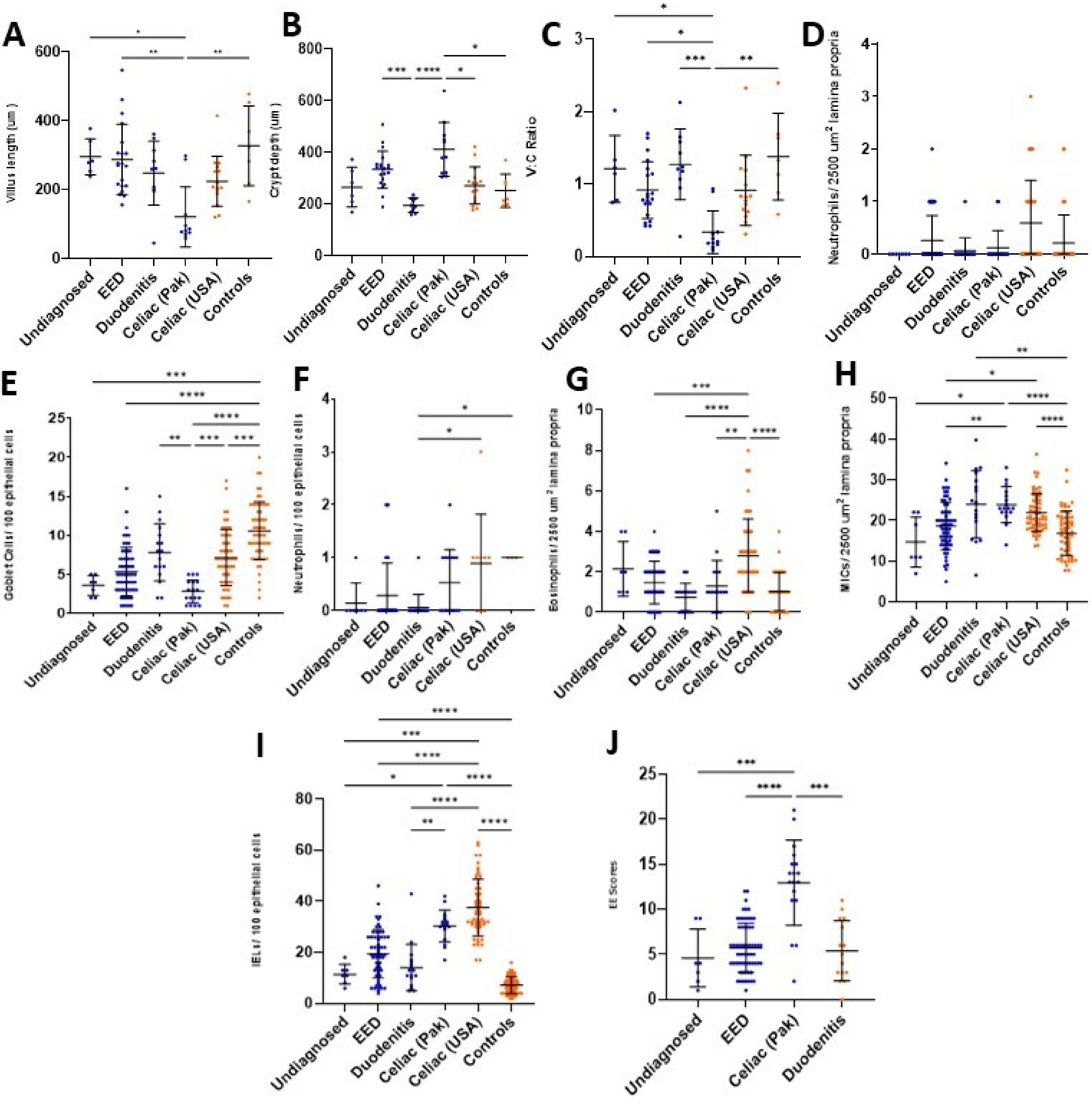
A
Villus length (um)
B
Crypt depth (um)
C
V:C Ratio
D
Neutrophils/ 2500 um² lamina propria
E
Goblet Cells/ 100 epithelial cells
F
Neutrophils/ 100 epithelial cells
G
Eosinophils/ 2500 um² lamina propria
H
MIICs/ 2500 um² lamina propria
I
IELs/ 100 epithelial cells
J
EE Scores
Undiagnosed
EED
Duodenitis
Celiac (Pak)
Celiac (USA)
Controls
